# Preparation and characterisation of NH_3_ gas sensor based on *PANI/Fe-doped CeO*_*2*_ nanocomposite

**DOI:** 10.1016/j.heliyon.2024.e34801

**Published:** 2024-07-20

**Authors:** Chakavak Esmaeili, Saeed Ashtiani, Chhabilal Regmi, Alexandr Laposa, Jan Voves, Jiří Kroutil, Karel Friess, Vojtech Povolny, Saeid Lotfian

**Affiliations:** a Czech Technical University in Prague, Faculty of Electrical Engineering, Technická 1902/2, 166 27, Prague 6, Czech Republic; b Department of Physical Chemistry, University of Chemistry and Technology Prague, Technická 5, 166 28, Prague 6, Czech Republic; c Ralph E. Martin Department of Chemical Engineering, University of Arkansas, Fayetteville, AR, 72701, United States; d Department of Naval Architecture, Ocean and Marine Engineering, University of Strathclyde, Glasgow, G4 0LZ, UK

**Keywords:** Polyaniline (PANI), Ceric oxide (CeO_2_), Fe doped nanocomposite, NH_3_ gas sensor, Interfering gases

## Abstract

*PANI/Fe-doped CeO*_*2*_*nanocomposite* was synthesised by the *in-situ* process. The produced powders were characterised by *XRD*, *XPS*, *FT-IR*, *Raman*, *HRTEM* and *SEM-EDS* tests. The sensors' function was based on *PANI/Fe-doped CeO*_*2*_*nanocomposite* with thin film deposited on top of interdigitated electrodes (*IDT*). *NH*_*3*_ detection with *PANI/Fe-doped CeO*_*2*_ nanocomposite sensor could be successfully performed even at room temperature (RT) and relative humidity of 45 %. Results demonstrated that *PANI/Fe-doped CeO*_*2*_ might be promising sensing materials for detecting the low *NH*_*3*_ concentration (ppm). In addition, the sensor is selective to the interfering gases, including *CO*, *CO*_*2*_ and *NO*_*2*_. This sensor displays acceptable repeatability and stability over time.

## 1 Introduction

The World Health Organisation (WHO) announced that 30.7 million people died in major cities from cancer chronic respiratory and cardiovascular diseases due to air pollution in the last five years. The health impacts of air pollution have received much attention in the detection of harmful pollutants in the atmospheric environment [1,2]. Ammonia (*NH*_*3*_), as a colourless gas, is harmful and foul-smelling gas, and it is one of the most abundant alkaline components in the atmosphere with a potent, pungent odour at ambient temperature [3]. Fertilisers, soils, and chemical manufacturing are some additional sources of *NH*_*3*_. Developing high-performance sensors to detect *NH*_*3*_ in the air rapidly and consistently appears highly necessary in this context. Besides the necessity of sensing low levels (ppm) of *NH*_*3*_, in some situations, such as the automatic management of the chemical manufacturing process, high levels (%) of *NH*_*3*_ are also required [4].

In recent decades, metal oxide-based gas sensors have received much attention. The existence of active sites on the metal oxide's surface is responsible for their gas-sensing properties. Furthermore, the mechanism of gas sensors mostly depends on the size of metal oxide particles and their crystallinity [5]. The intrinsic properties of metal oxide, namely low cost, thermal stability, nontoxicity, and high chemical sensitivity, are ascribed to high-density free charge carriers [6]. Ceric oxide (*CeO*_*2*_) is one of the most used oxides for developing detectors of toxic gases, such as CO, NOx, NH3, and hydrocarbons. *CeO*_*2*_-based material is an n-type semiconductor with an energy band gap of 3.19 eV [7]. Applications of *CeO*_*2*_ to gas sensor detection have also attracted considerable interest due to the generation of lattice defects with cubic fluorite, distinct physical and chemical features with outermost 4f shell, and high oxygen storage capacity with low cost [8]. Moreover, the low redox potential between Ce^+3^ and Ce^+4^ has made this oxide an advantageous sensing material for detecting gases. Furthermore, researchers have demonstrated that *CeO*_*2*_ is viable for detecting explosive, toxic, volatile organic compounds (*VOCs*) and hazardous gases [[9], [10], [11]]. The most crucial characteristic of ceria is the capacity to store and release oxygen via facile Ce^+4^/Ce^+3^ redox cycles [12]. *CeO*_*2*_ is known as an insulator; thus, due to its ionic conductivity by introducing oxygen vacancies in the lattice as charge-compensating defects, doping with different rare earth elements, alkaline cations, and/or transitional metals has been reported [13].

The introduction of noble metal nanostructures (e.g., Au, Ag, Al, Fe) has been considered an effective gas detection method [14,15]. Similarly, the cubic fluorite structure of pristine *CeO*_*2*_ can support the stoichiometric deviations, which, thus, proves to be advantageous when *CeO*_*2*_ is doped with a small fraction of transition metal ions like Fe. Such a procedure is anticipated to enhance their properties without distorting the original structure. Among different dopants, Fe is attractive and environmentally friendly as a dopant of *CeO*_*2*_ because it can improve the catalytic activity due to its redox ability since the oxygen species can alter between Fe^3+^/Fe^2+^ [16,17]. In addition, the amounts of Fe dopant on the humidity-sensing properties of *CeO*_*2*_ *NPs* were validated by humidity-sensing studies [18]. Essentially, the poorer electrical conductivity of metal oxides at ambient temperature limits their gas-detecting effectiveness [19]. To solve this problem, superior gas sensing results can be obtained by combining *CeO*_*2*_ with other materials such as ZnO, nanocrystals, graphene, WO_3_ and modifying their structure [10,20]. Low-dimensional nanomaterials and polyaniline (*PANI*) in nanocomposites produce outstanding synergies [21,22]. Adsorption of gas molecules promotes de-doping of *PANI* particles, which alters the diameter of the space charge zone of restricted heterojunctions [23]. The porous and loose nanomaterial structure could provide vast anchor sites to adhere to a *PANI* as a conductive substrate.

## 2 Experimental section

### 2.1 Materials and instruments

Ammonium persulfate (98 %), cerium nitrate hexahydrate (Ce(NO_3_)_3_·6H_2_O)(99.99 %), ferric (III) nitrate nanohydrate (Fe(NO_3_)_3_·9H_2_O)(98 %), sodium hydroxide (NaOH) were purchased from Sigma-Aldrich, and aniline was obtained from Penta (Czech Republic). All chemicals were used as received without further purification. IDT electrodes (Au/Cu interdigital) were purchased from Pragoboard company (Czech Republic). A sonicator bath (Jeken) and a vortex (Verkon) were used to prepare homogenous mixtures. Deionised water was utilised to prepare all of the aqueous solutions.

### 2.2 Material characterisation

Scanning electron microscope (SEM), Tescan LYRA, equipped with energy dispersive spectroscopy (EDS), Oxford Instruments, 80 mm^2^, was used for morphology determination. The EDS was utilised for chemical microanalysis of elements present and/or comprehensive analysis of element distributions within the materials. The secondary electron detector measured samples themselves while the accelerating voltage was 15 kV. Powders were placed on a double-sided adhesive tape made of carbon and covered by 2 nm of Au to ensure their excellent conductivity. Transmission electron microscopy (TEM) analysis of the synthesised nanoparticles/nanocomposite was performed using a JEM-2200FS Jeol instrument. Fourier transform infrared spectroscopy (FTIR) measurements were performed on an iS50R FTIR spectrometer (Thermo Scientific). The measurement was performed using a DLaTGS detector and KBr beam splitter in 4000-500 cm^−1^ at a resolution of 4 cm^−1^. X-ray diffraction (XRD) measurement was performed using 2nd Generation D2 Phaser X-ray diffractometer (Bruker) with Cu Kα radiation (λ = 0.15418 nm), SSD (1D mode) detector, coupled 2θ/θ scan type and continuous PSD fast scan mode. The range of measured Bragg 2θ angle was from 5 to 80°. High-resolution X-ray photoelectron spectroscopy (XPS) measurement was performed using an ESCAProbeP Spectrometer (Omicron Nanotechnology Ltd.) with a monochromatic aluminium X-ray radiation source (1486.7 eV). Raman spectroscopy measurements were performed on a Renishaw via Raman microscope using a 532 nm laser in a backscattering geometry with a Charge Coupled Device (CCD) detector.

### 2.3 Synthesis of *Fe-doped CeO*_*2*_ nanocomposite

The 0.01 mol of Ce(NO_3_)_3_·6H_2_O was dissolved in 100 ml of deionised (DI) water with constant stirring. NaOH (5 M) was added to the solution dropwise with constant stirring until complete precipitation. The precipitate was then stirred for 3 h, followed by hydrothermal treatment using an autoclave, maintaining the temperature of 110 °C for 24 h. The nanoparticles were washed several times with water and finally with ethanol using centrifugation. The nanoparticles were dried in an oven overnight at 80 °C followed by calcination at 500 °C for 3 h. For the synthesis of 7 mol % Fe doped *CeO*_*2*_ nanocomposite, 0.282 g of Fe(NO_3_)_3_·9H_2_O was added to the aqueous solution of Ce(NO_3_)_3_·6H_2_O prior to the addition of NaOH solution and followed the similar protocol described above.

### 2.4 Preparation of *PANI/Fe-doped CeO*_*2*_ nanocomposite layers

*PANI* in the form of protonated emeraldine salt was synthesised by oxidising 0.2 M aniline hydrochloride with 0.25 M ammonium persulfate at room temperature (RT), as described in the literature [24]. An exothermic reaction occurred during the *PANI* synthesis, and the temperature of the reaction mixture was checked. The polymerisation process was completed for 15 min at 37 °C with gentle stirring. The dark green precipitate was filtered off and washed with acetone and 0.2 M hydrochloric acid several times. Afterwards, the *PANI* was dried in a desiccator overnight. The *PANI/Fe-doped CeO*_*2*_ nanocomposite was prepared by mixing 2 mg *Fe-doped CeO*_*2*_ and 10 mg PANI in 1 mL xylene.

The *PANI* with *Fe-doped CeO*_*2*_ nanocomposite suspension was ultra-sonicated for 2.0 h and then gently mixed by a vortex device for approximately 1 h.

### 2.5 Gas sensing measurements

All gas sensing studies were conducted in the RT gas chamber (27 °C). A Keithley 2400 source meter was used to measure the current versus time characteristics at a constant DC input voltage of 1 V.

**Fig. 1:**
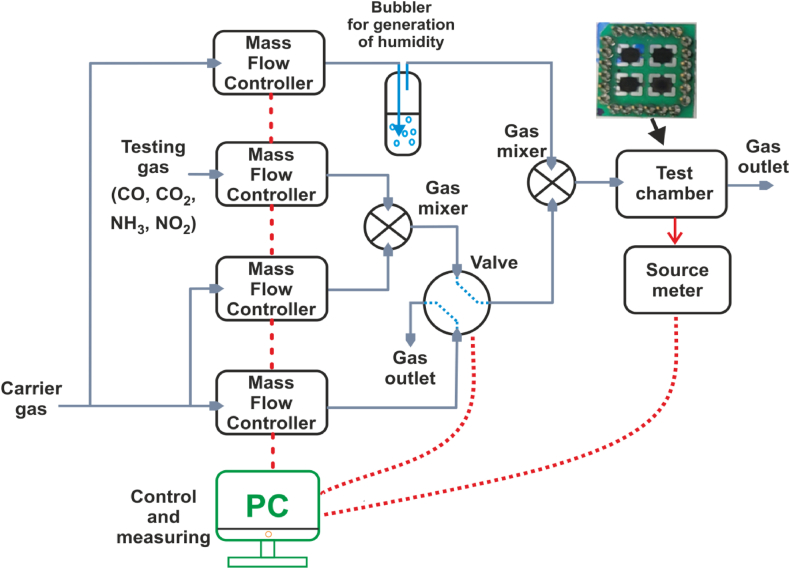
Schematic diagram of the gas sensing characterisations apparatus.

Electrical feed through the *NH*_*3*_ gas sensor was placed into an airtight testing chamber. With a specific gas concentration at RT, the resistance of the *NH*_*3*_ gas sensor was continually recorded by a computer. In this work, the response of the gas sensor is defined by the ratio of (Rg−Ra)/Ra for the testing of *NH*_*3*_ gas, where R_a_ is the resistance of the sensor with synthetic air dilution, and R_g_ is the sensor resistance in pollutant target gas. In addition, the total gas flow rate into the chamber was maintained at 200 ml per minute. A schematic diagram of the characterisation of the gas sensor is shown in Fig. 1.

## 3 Results and discussions

### 3.1 Characterisation of *PANI/Fe-doped CeO*_*2*_ nanocomposite

a) *XRD, Raman Spectroscopy, FT-IR*

**Fig. 2:**
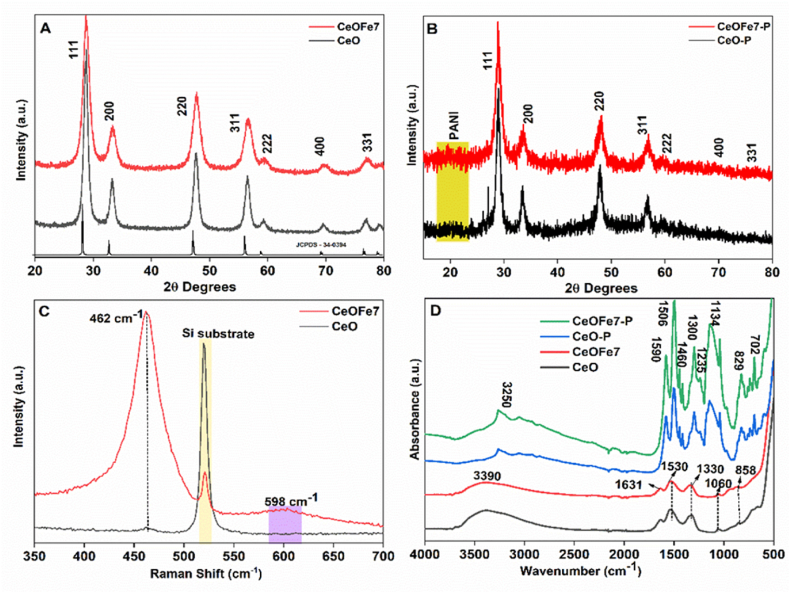
A) XRD of undoped and *Fe-doped CeO*_*2*_*nanocomposite*; B) XRD of the *nanocomposite* @*PANI* composites; C) Raman spectra of *nanocomposite*; D) FTIR of the pristine *nanocomposite* and nanoparticles @*PANI* composites.

Fig. 2A shows the XRD spectra of pure *CeO*_*2*_ and *Fe–doped CeO*_*2*_ nanocomposite samples. The diffraction peaks at the corresponding 2θ values well matched with the JCPDS -34-0394 for every *CeO*_*2*_ sample, confirming the fluorite structured *CeO*_*2*_ with Fm3m space group [25]. The strong diffraction peaks indicated the good crystalline nature of the samples. No noticeable change in the diffraction patterns, i.e., any additional peaks related to Fe dopant, were detected, signifying the single phase, highly pure nature, and proper substitution of Fe ion at the Ce site in the nanocrystals. These further suggest the complete dissolution of Fe into the ceria lattice and the formation of a solid solution of *Ce–O–Fe*. The appearance of a broad peak centred at 19.5 2θ value that corresponds to the peak of PANI as well as the characteristics peaks of *CeO*_*2*_ nanocomposite at corresponding 2θ values with diminished peak intensity (Fig. 2B) representing lower crystallinity confirms the formation of *PANI/Fe-doped CeO*_*2*_ nanocomposite.

Raman spectra of the synthesised nanocomposite (Fig. 2C) exhibit a single active mode centred at 462 cm^−1^, characteristics of the cubic fluorite ceria phase [26]. An additional broad peak centred at 598 cm^−1^ was also observed in the Fe-doped samples. This peak is attributed to the oxygen vacancy and disturbance of the local symmetry induced by dopants [27,28]. This also signifies the homogeneous incorporation of Fe within the CeO_2_ crystal structure. A peak centred at 520 cm^−1^ is attributed to the Si substrate.

Fig. 2D shows the *FTIR* spectra of undoped, *Fe-doped CeO*_*2*_ nanocomposite and *CeO*_*2*_ @ *PANI* composite. In the case of nanocomposite samples, the intense absorption band at 3390 and 1516-1630 cm^−1^ are associated with symmetrical stretching and bonding mode of internally bonded water molecules (O–H), respectively. The O–C–O stretching band observed in 1330 cm^−1^ and 1060 cm^−1^ regions confirms the surface adsorbed CO_2_. The absorption band at 858 cm^−1^ is produced by *CeO*_*2*,_ a typical peak for Ce–O stretching vibration [29]. Similarly, in the case of composite samples, the peaks at 1460, 1235, and 702 cm^−1^ agree with the functional groups of *PANI*. The peaks in the 1590 cm^−1^ region are assigned to the stretching of quinonoid whereas, at 1506 cm^−1,^ assigned to the C–C stretching mode of the benzeneoid ring, 1300 cm^−1^ is assigned to C–N stretching mode, and 1134 cm^−1^ is assigned to C=N stretching of secondary aromatic amine. The N–H stretching vibration of aromatic amines is assigned at 3250 cm^−1^ [30,31]. No prominent peaks for *CeO*_*2*_ could be observed in the composite samples. The dominance of the *PANI* signature peaks in the composite samples indicates the *PANI* matrix's encapsulation of the *CeO*_*2*_ oxides during the synthesis process. Moreover, the slight shifting of peaks to lower wavenumbers compared to pure *PANI* [32] is presumed due to hydrogen bonding between the hydroxyl groups on the surface of the *CeO*_*2*_ nanoparticles and the imine groups in the *PANI* molecular chain [33].

b) XPS

**Fig. 3:**
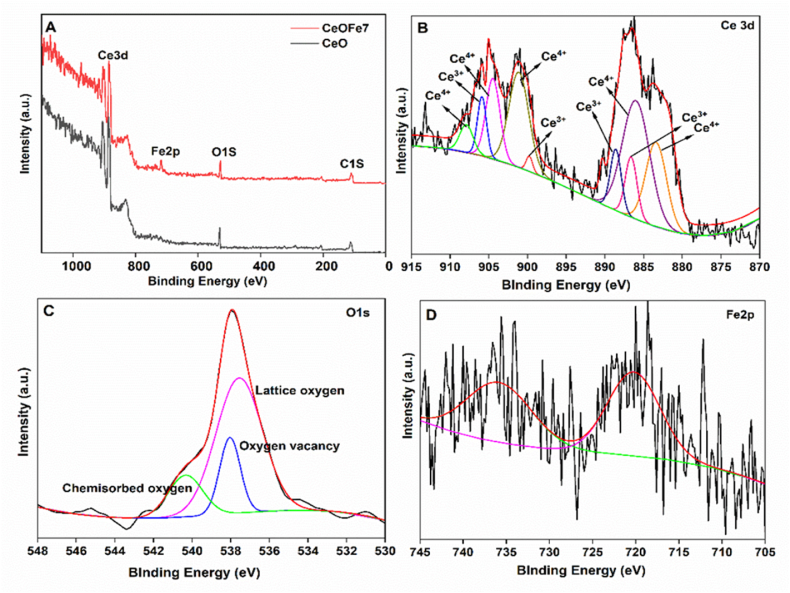
XPS of undoped and *Fe doped CeO*_*2*_ nanoparticles in the study; A) Survey spectrum of undoped and *Fe doped CeO*_*2*_ nanoparticles; B), C0 and D) are the high-resolution spectra of Ce3d, O1s and Fe 2p respectively.

Wide scan XPS spectrum (Fig. 3A) exhibits evident *CeO*_*2*_ features and the presence of an additional Fe 2p signal in the 705–745 eV range, indicating that the Fe particles had been successfully incorporated into the *CeO*_*2*_ matrix. The fitted Ce 3d spectra of the *Fe-doped CeO*_*2*_ sample (Fig. 3B) showed different peaks corresponding to different oxidation states of Ce^+3^ and Ce^+4^. The presence of Ce^+3^ reveals the presence of oxygen vacancies in the samples. The presence of the Ce^+3^ state is due to the reduction of Ce^+4^ in the oxide structure. Thus, oxygen vacancies are presumed to be produced due to electron transformation between Ce^+3^ and Ce^+4^ [34]. The existence of Ce^+3^ is a direct consequence of the presence of the Ce–*O*–Fe bridges on the surface. The charge compensation by Fe insertion makes part of Ce^+4^ transformation into Ce^+3^ associated with forming oxygen vacancies and lattice defects favorable for oxygen mobility [35,36]. The O1s region (Fig. 3C) contained three contributions, one due to lattice oxygen with the binding energy of 537 eV, the second peak attributed to chemisorbed oxygen species on the surface (OOH), with the binding energy of 538 eV arising due to dissociative adsorption of water. In contrast, the third peak with the binding energy of 540 eV is attributed to oxygen vacancy. The slight shifting of the peak position of oxygen is attributed to the incorporation of Fe ions in the CeO_2_ lattice [26,37]. In Fe2p spectra (Fig. 3D), diminished peaks of Fe2p detected at around 738 eV and 721 eV indicate low Fe^+3^ content [38].

c) SEM, TEM, HRTEM

**Fig. 4:**
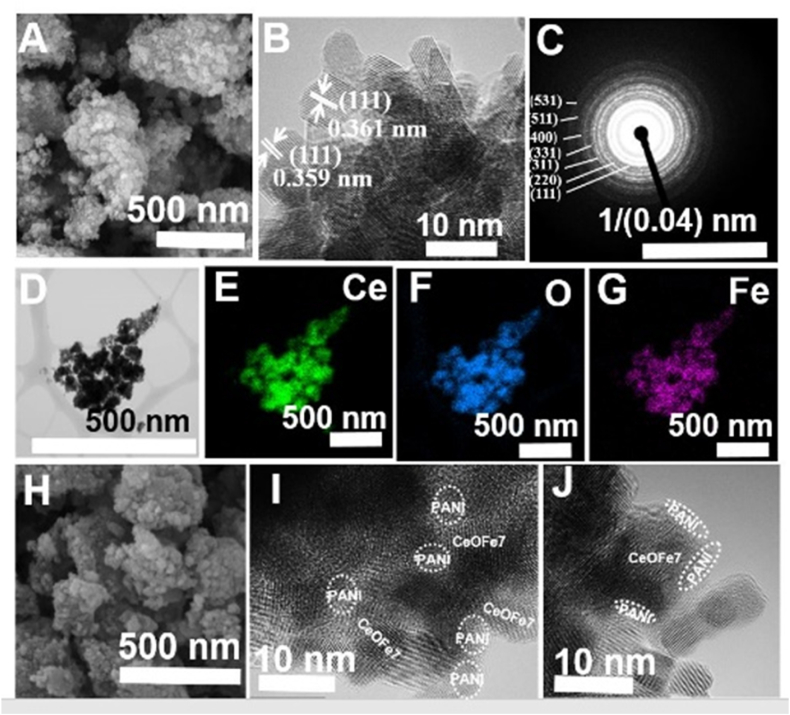
A) SEM image, B) HRTEM image, C) SAED pattern, D) TEM image, (E–G) EDS elemental mapping of Fe doped CeO_2_ nanoparticle and H) SEM image, (I–J) HRTEM image of the CeOFe7@PANI composite particles.

Figure S1 (A) and (B) are the SEM images of undoped *CeO*_*2*_ *NPs* and *CeO*_*2*_*@PANI* composite, showing agglomeration of the synthesised particles. Figures S1 (C) and (D) are the typical high-resolution TEM (HRTEM) image and selected area electron diffraction (SAED) analysis of CeO_2_ nanoparticles. The SAED analysis reveals that the prepared *CeO*_*2*_ nanoparticles display typical polycrystalline rings, and the clear lattice fringes with a space of about 0.362 ± 0.003 nm can be observed corresponding to the interplanar distances of (111) of cubic fluorite *CeO*_*2*_ [39]. Similarly, Fig. 4 (A) shows the SEM image of the *Fe-doped CeO*_*2*_ sample. The overall morphology of *Fe-doped CeO*_*2*_ is similar to that of undoped *CeO*_*2*_ *NPs*. Similar to *CeO*_*2*_ nanoparticles, the HRTEM image (Fig. 4B) reveals the nanocrystalline domain in the Fe-doped *CeO*_*2*_ sample. Due to the formation of mixed solutions, phase segregation of Fe and *CeO*_*2*_ is not observed, which is further supported by the XRD pattern. The slight decrease in the interplanar distances (0.360 ± 0.001 nm) revealed the shrinkage of the unit cell as a result of doping metal ion (Fe) with a smaller radius into the *CeO*_*2*_ lattice [38]. Selected area electron diffraction patterns of both undoped (Figure S1 D) and *Fe-doped CeO*_*2*_ samples (Fig. 4C) displayed similar diffraction rings demonstrating single-phase and polycrystalline nature. The inner to outer diffraction rings can be indexed to the (111), (220), (311), (331), (400), (511) and (531) plane of *CeO*_*2*_ (JCPDS - 34–0394) consistent with XRD patterns. The TEM micrograph of the *Fe-doped CeO*_*2*_ sample (Fig. 4D) also shows moderate agglomeration of the NPs with quasi-spherical shapes. Elemental analysis of the *Fe-doped CeO*_*2*_ sample (Fig. 3E–G) showed the presence of Ce, O and Fe, which further confirms the successful doping of Fe into the CeO_2_ lattice. Fig. 4H is the SEM image of the *CeOFe7@PANI* composite. Agglomerated non-uniform structures of composite particles were observed. The HRTEM images of the composite (Fig. 4I–J) depict that nanoparticles (*Fe doped CeO*_*2*_) are surrounded by the *PANI* matrix, forming a core-shell-like structure, which thus reveals the attachment of *PANI* to CeOFe7 nanoparticles. This suggests that blending the conductive nature of the *PANI* network with the Ceria nanoparticles leads to the interaction between the polymer particles and the nano ceria, ultimately forming a composite of *CeOFe7@ PANI*. Similarly, *PANI*, which has a large number of functional groups, provides sites for unbound *CeO*_*2*_ nanoparticles through self-attachment [40].

### 3.2 NH3 sensing performance based on *PANI/Fe-doped CeO*_*2*_ nanocomposite

**Fig. 5:**
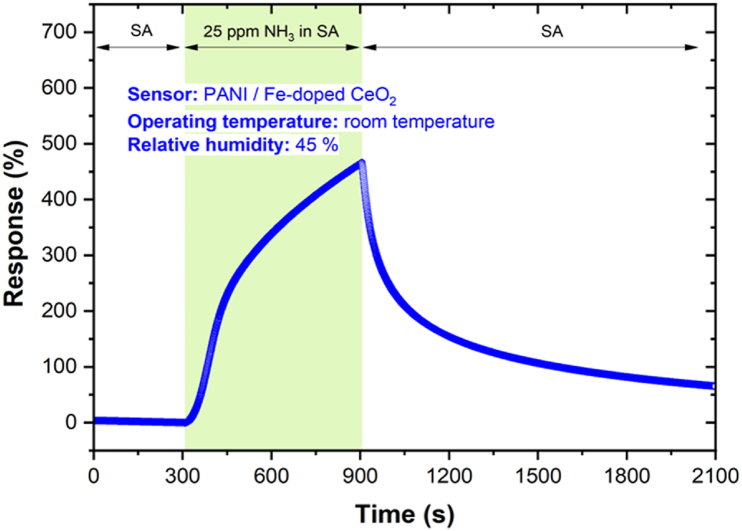
Response of the *PANI/Fe-doped CeO*_*2*_ nanocomposite to 25 ppm of *NH*_*3*_ at RT and RH 45 %.

The abovementioned SEM result clearly indicates that the *PANI/Fe-doped CeO*_*2*_ could be described as a large specific surface of nanoparticles and multiple gaps. This result is increased gas molecule adsorption sites, and adequate time for gas molecule adsorption and diffusion saturation may be required [41]. The sensors' responses upon exposure to 25 ppm NH_3_ at RT is shown in Fig. 5. A small amount of surface interacts with the air molecules in dark conditions, causing negligible response change. The response increases the chemical activity of the surface by enhancing the number of charge carriers in the conduction band as long as there is a higher number of active sites on the surface [42]. Hence, it improves the adsorption capacity concentration on the surface by providing a higher number of electrons. When air is introduced to the nanoparticles, oxygen is absorbed molecularly at RT. The *NH*_*3*_ gas molecules react with the excited electrons/holes and adsorbed oxygen ions and turn into the products (Fig. 5). In addition, the *CeO*_*2*_ *NPs* alone might block the charge carriers or reduce the delocalisation length and hence increase the resistance of the nanohybrid when exposed to *NH*_*3*_ gas [43]. Our previous study has shown that the PANI composites' responses saturate at a higher gas concentration. It could be due to a reduced surface area with possible reaction sites on the surface of the film [24].

It could be considered that when the *PANI/Fe-doped CeO*_*2*_ nanocomposite was exposed to *NH*_*3*_ gas, the *NH*_*3*_ molecules would diffuse into the nanocomposite surface, and *PANI* can cause swelling, thereby increasing the interchain distance and thus decreasing the conductivity of *PANI* [41]. The capability of *CeO*_*2*_ to retain oxygen through a unique redox reaction between Ce^3+^ and Ce^4+^ ions will be critical in gas detection. The doping of Fe increases oxygen vacancy and improves gas-sensing behaviour [44]. The creation of oxygen vacancy not only enhances the oxygen storage capacity of the materials but also enhances the efficiency of the surface in reacting with the surrounding environment [13].

Since the Ce^3+^ ions can exhibit oxygen vacancies with two units of negative charges (V_O_), the content of Ce^3+^ ions affect the amount of oxygen vacancies. The higher VO concentration typically causes more chemisorbed oxygen molecules on the *CeO*_*2*_ surface, providing enough active sites for gas molecules and allowing more gas molecules to be adsorbed on the *CeO*_*2*_-based sensor's surface [8]. As a result, oxygen vacancies improve gas responsiveness [45]. The development of an oxygen defect is accompanied by the localisation of electrons left in Ce 4f states, causing the formation of two Ce^3+^ ions while maintaining the cubic fluorite crystal structure (Eq (1)).

(1) 2 Ce^4+^ +O^2−^ ↔ 2 Ce^3+^ + V_O_ + 1/2 O_2_

The surface of V_O_ can be an electron donor, and more electrons would flow from the *PANI-CeO*_*2*_ surface to the Fe-doped surface, directly enhancing conductivity. As for the pure CeO_2_ nanoparticles, electrons would be trapped by Ce^4+^.

When the sensor is put back into the air, the electrons combine with oxygen on the surface again, and the following Eq. (2) will occur [46]:

(2) [ 2 Ce^3+^, V_O_ ] + O_2_ → [ Ce^4+^, Ce^3+^, O^2−^ ]

*Fe-doped CeO*_*2*_ systems present a remarkable improvement in their oxygen exchange abilities compared to the pristine *CeO*_*2*_ because of the Ce–Fe synergy that is achieved by combining the redox behaviour of the Ce^+4^/Ce^+3^ and Fe^+3^/Fe^+2^ cations [47]. When transition metal ion Fe^+2^/Fe^+3^ is doped into *CeO*_*2*_, it substitutes Ce^+4^ and liberates oxygen, which may take the position in interstitial lattice sites probably due to the smaller ionic state as well as the ionic size of Fe^+2^(0.74A)/Fe^+3^(0.78A) as compared to that of Ce^+4^ (0.97A) [13].

A concentration lower than 7 mol% of iron exhibited negligible response, and high concentration of 7 mol % Fe increased resistance and then higher adsorption capacity for Fe, which confirms that *PANI/Fe-doped CeO*_*2*_ is more difficult to transport through media.

**Fig. 6:**
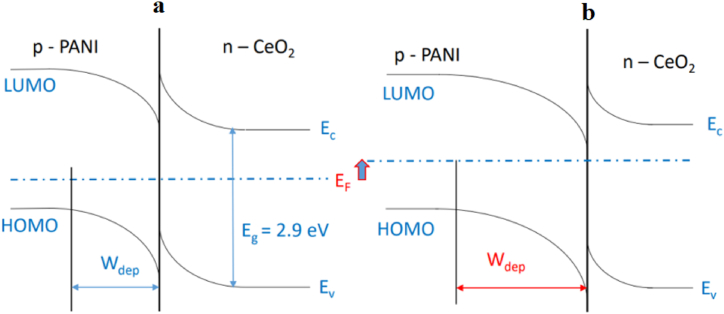
(a) Schematic band structure of the *PANI/CeO*_*2*_ interfaces in air and (b) in the presence of *NH*_*3*_.

The resistance of the *CeO*_*2*_, *PANI/Fe-doped CeO*_*2*_ composite sensor increases, whereas the resistance of the *PANI* sensor decreases due to changes in the depletion layer widths, as shown in Fig. 6. Hence, the as-prepared *PANI* with *Fe-doped CeO*_*2*_ nanocomposite behaves like a p-n junction material which was evident from the positive Seebeck coefficient values, while *CeO*_*2*_ and *PANI* are promoting n-type and p-type charge, respectively. When the sensor is switched to air, the resistance will revert to its base value with good reversibility of the sensor [48]. The improvement of protonation degree and modified morphology of *PANI* by the addition of metal oxide nanoparticles due to the unique *p-n* junction between *PANI* and gas causes the excellent performances of sensor selectivity based on nanocomposite [24].

**Fig. 7:**
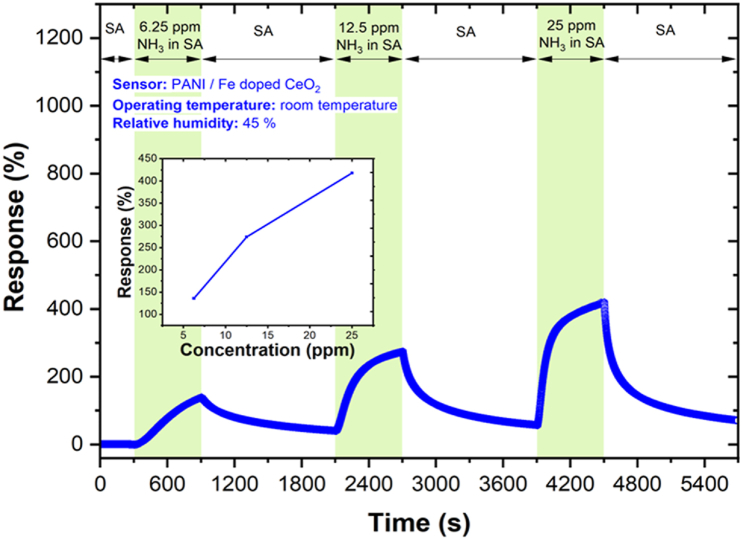
Dynamic response of *NH*_*3*_ gas sensor and related calibration curve. The response towards 6.25, 12.5 and 25 ppm of *NH*_*3*_ gas at RT.

**Fig. 8:**
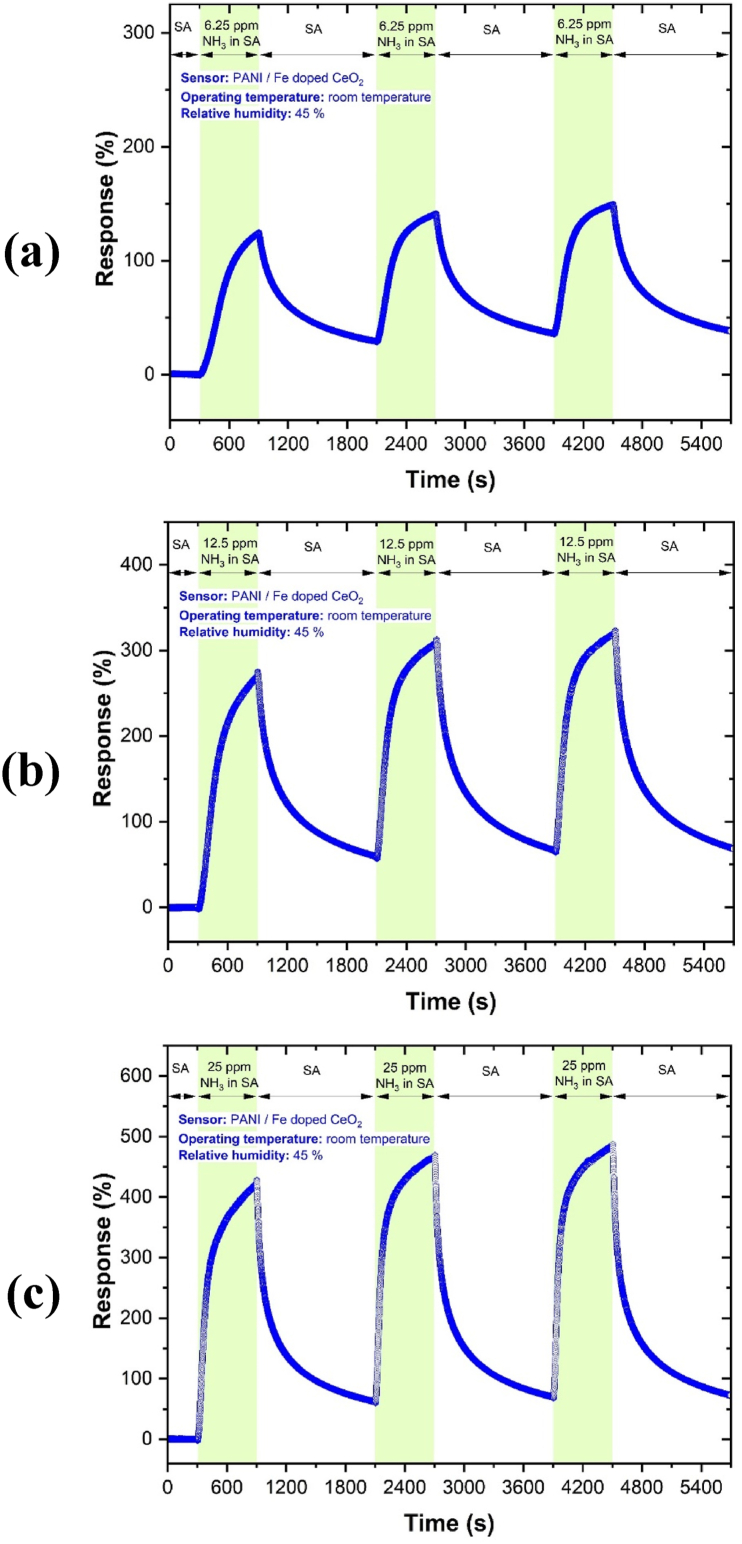
Repeatability of the transient response of *NH*_*3*_ gas sensor to a) 6.25, b) 12.5 and c) 25 ppm *NH*_*3*_ at RT.

The sensing results towards 6.25, 12.5 and 25 ppm concentrations of *NH*_*3*_ exhibited promising behaviour (Fig. 7). The increased response in the sample with *PANI/Fe-doped CeO*_*2*_ nanocomposite heterojunction is attributed to the new electronic interface states. The repeatability result of the gas sensor for 6.25, 12.5 and 25 ppm *NH*_*3*_ at RT is shown in Fig. 8, which indicates the *PANI* with *Fe-doped CeO*_*2*_ nanocomposite has good repeatability. The results confirm the repeatability of the present sensor to sequential exposures to NH_3_ gas to the mean output of all three exposures (6.25, 12.5 and 25 ppm concentrations of *NH*_*3*_*)*.

Due to determining the sensor's stability toward 25 ppm *NH*_*3*_ over a more extended period, the performance of *Fe-doped CeO*_*2*_ nanocomposite after 6 months was lowered to 87.6 %. In 6 months, the response has changed slightly.

**Fig. 9:**
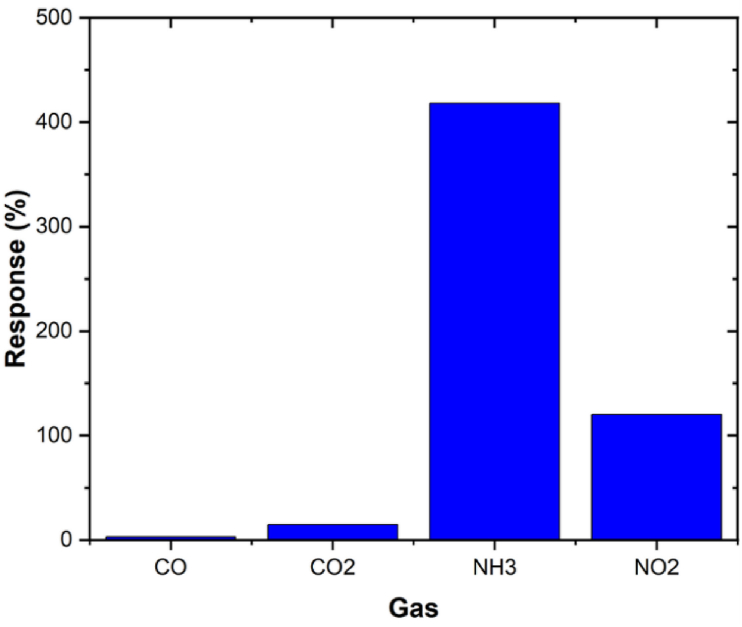
Four gases selectivity of the sensors based on *PANI/Fe-doped CeO*_*2*_ nanocomposite, operating condition: (25 ppm of each gas concentration at RT and RH 45 %).

Selectivity is one of the essential parameters in the study of gas sensors. Theoretically, in the same controlled environment, a sensor that is extremely sensitive to one gas is less sensitive to other gases. Sensors, with *PANI/Fe-doped CeO*_*2*_ nanocomposite as the sensing layer, were investigated for various gases, such as *CO*, *CO*_*2*_, and *NO*_*2*_, at ambient temperature. When the sensor is exposed to 25 ppm of various gases, including *CO*, *CO*_*2*_, *NH*_*3*_, and *NO*_*2*_ at the ambient temperature, the *PANI/Fe-doped CeO*_*2*_ nanocomposite is most sensitive to *NH*_*3*_ (Fig. 9). The low selectivity of *CO* and *CO*_*2*_ can be attributed to the crystallite's size and the surface *PANI/Fe-doped CeO*_*2*_'s relative concentration. The selectivity of the PANI/Fe-doped CeO_2_ composite sensor towards NH_3_ over CO, CO_2_, and NO_2_ is primarily due to the specific surface interactions and adsorption energies. NH3 molecules form stronger interactions with the surface functional groups of PANI/Fe-doped CeO_2_, resulting in higher adsorption energy and a more pronounced change in the sensor's electrical properties. The crystallite size and relative surface concentration of PANI/Fe-doped CeO_2_ enhance this effect by providing a higher surface area and more active sites for NH_3_ adsorption.

Furthermore, the surface functional groups, such as amino and hydroxyl groups, present on the composite material create selective binding sites for NH_3_ through strong hydrogen bonding, which is less favorable for CO, CO_2_, and NO_2_. This selective adsorption mechanism explains why the sensor exhibits a higher response to NH_3_ compared to other gases.

The gas sensing results against 25 ppm *NH*_*3*_ indicated that the response of *NH*_*3*_ is almost 4 times greater than that of the other gas samples. *NH*_*3*_ gas sensing mechanism is measured by electrical conductivity in relation to an air atmosphere baseline. Oxygen species that are absorbed from the air onto the surface of *CeO*_*2*_ materials can be ionised to absorb oxygen ions (O_x_^−^) by absorbing the free electrons from the sensing element (Eqs (3), (4), (5), (6))) [49]. The following equation can be used to explain this process:

(3) O_2gas_↔O_2ads_

(4) O_2ads_ + e^−^↔O^−^_2ads_

(5) O^−^_2ads_ + e^−^↔2O^−^_ads_

(6) O^−^_ads_ + e^−^↔O^2−^_ads_

After ammonia is introduced to the gas sensor element, the ammonia gas reacts with the oxygen ions that have been adsorbed, releasing the captured electron and resulting in a lower barrier potential and thinner space charge (Eq (7)) [50].

(7) 4*NH*_3_+3*O*_2_^−^_*ads*_↔2*N*_2_+6*H*_2_*O*+3*e*^−^

When ammonia interacts with the oxygen adsorbed on the surface, trapped electrons are released onto the surface, increasing the surface conductivity in *PANI/Fe-doped CeO*_*2*_ nanocomposite [51].

This is mainly in the presence of many surface defects due to its high surface area and the higher bulk density of charge carriers, which enable the sensor to absorb more gas molecules [52]. The long response time of *NH*_*3*_ can be attributed to the longer migration path of gas molecules to reach the active areas within the *PANI/Fe-doped CeO*_*2*_ nanocomposite s' structure [53]. Table 1, summarises the comparison of the developed *NH*_*3*_ performance with other materials.

**Table 1:** Comparison of sensing performance of various materials toward *NH*_*3*_ gas at RT.

Material	Fabrication Method	Response	Ref
*PANI/Fe-doped CeO*_*2*_*nanocomposite*	in situ polymerisation	500 % towards 25 ppm NH_3_	This work
*PANI-CeO*_*2*_*nanocomposite*	in-situ self-assembly	262.7 % towards 50 ppm NH_3_	[41]
*NiO sphere- PANI*	solvothermal	43 % towars 10 ppm NH_3_	[54]
*Camphor Sulphonic Acid (CSA)/PAni-CeO*_*2*_	facile chemical oxidative polymerisation	93 % towards 100 ppm NH_3_	[55]
*CeO*_*2*_*nanoparticles*	Hydrothermal	∼350 % towards 25 ppm NH_3_	[56]
*PANI-iron oxide nanocomposite*	in-situ polymerisation	39 % towards 100 ppm NH_3_	[57]
*Fuzzy nanofibrous network of PANI film*	in situ method	∼35 % towards 50 ppm NH_3_	[58]

## 4 Conclusion

In summary, the PANI/Fe-doped CeO_2_ nanocomposite has been fabricated by in situ polymerisation of PANI in the presence of CeO_2_ NPs. PANI with Fe-doped CeO_2_ was characterised by various techniques and investigated for NH_3_ gas detection at room temperature. The performance of the NH_3_ gas sensor is considered an effect of pn-junction, the enhanced degree of protonation and its modified morphology of PANI due to the addition of Fe-doped CeO_2_ nanocomposite. The selectivity of the gas sensing system was successfully tested on four different gases with responses to 6.25, 12.5 and 25 ppm with low concentrations of NH_3_ and high repeatability.

## CRediT authorship contribution statement

**Chakavak Esmaeili:** Writing – review & editing, Writing – original draft, Validation, Project administration, Investigation. **Saeed Ashtiani:** Writing – review & editing. **Chhabilal Regmi:** Writing – original draft, Methodology, Investigation. **Alexandr Laposa:** Writing – review & editing, Investigation. **Jan Voves:** Validation, Project administration. **Jiří Kroutil:** Methodology. **Karel Friess:** Project administration. **Vojtech Povolny:** Investigation. **Saeid Lotfian:** Project administration.

## Declaration of competing interest

The authors declare that they have no known competing financial interests or personal relationships that could have appeared to influence the work reported in this paper.

## References

[1] Sun Y., Zhang Y. (2023). Wafer-scale floating-gate field effect transistor sensor built on carbon nanotubes film for Ppb-level NO2 detection. Chem. Eng. J..

[2] Zhan S., Zuo H., Liu B., Xu W., Cao J., Zhang Y., Wei X. (2023). Wafer-Scale field-effect transistor-type sensor using a carbon nanotube film as a channel for ppb-level hydrogen sulfide detection. ACS Sens..

[3] Liu M., Wang J., Song P., Ji J., Wang Q. (2022). Metal-organic frameworks-derived In2O3 microtubes/Ti3C2Tx MXene composites for NH3 detection at room temperature. Sensor. Actuator. B Chem..

[4] Fu T. (2009). Research on gas-sensing properties of lead sulfide-based sensor for detection of NO2 and NH3 at room temperature. Sensor. Actuator. B Chem..

[5] Li Q., Zeng W., Li Y. (2022). Metal oxide gas sensors for detecting NO2 in industrial exhaust gas: recent developments. Sensor. Actuator. B Chem..

[6] Venkateshaiah A., Černík M., Padil V.V. (2022). Metal oxide nanoparticles for environmental remediation. Nanotechnol. Environ. Remediation.

[7] Deshmukh K., Pasha S.K. (2020). Room temperature ammonia sensing based on graphene oxide integrated flexible polyvinylidenefluoride/cerium oxide nanocomposite films. Polymer-Plastics Technol. Mat..

[8] Oosthuizen D., Motaung D., Swart H. (2020). Gas sensors based on CeO2 nanoparticles prepared by chemical precipitation method and their temperature-dependent selectivity towards H2S and NO2 gases. Appl. Surf. Sci..

[9] Dong Z., Hu Q., Liu H., Wu Y., Ma Z., Fan Y., Li R., Xu J., Wang X. (2022). 3D flower-like Ni doped CeO2 based gas sensor for H2S detection and its sensitive mechanism. Sensor. Actuator. B Chem..

[10] Hui G., Zhu M., Yang X., Liu J., Pan G., Wang Z. (2020). Highly sensitive ethanol gas sensor based on CeO2/ZnO binary heterojunction composite. Mater. Lett..

[11] Liao L., Mai H., Yuan Q., Lu H., Li J., Liu C., Yan C., Shen Z., Yu T. (2008). Single CeO2 nanowire gas sensor supported with Pt nanocrystals: gas sensitivity, surface bond states, and chemical mechanism. J. Phys. Chem. C.

[12] Wang B., Zhu B., Yun S., Zhang W., Xia C., Afzal M., Cai Y., Liu Y., Wang Y., Wang H. (2019). Fast ionic conduction in semiconductor CeO2-δ electrolyte fuel cells. NPG Asia Mater..

[13] Kumar S., Alharthi F.A., Ahmed F., Ahmad N., Chae K., Kumari K. (2021). Role of Fe doping on surface morphology, electronic structure and magnetic properties of Fe doped CeO2 thin film. Ceram. Int..

[14] Sun Y., Hu J., Zhang Y. (2022). Visible light assisted trace gaseous NO2 sensor with anti-humidity ability via LSPR enhancement effect. Sensor. Actuator. B Chem..

[15] Chen X., Hu J., Chen P., Yin M., Meng F., Zhang Y. (2021). UV-light-assisted NO2 gas sensor based on WS2/PbS heterostructures with full recoverability and reliable anti-humidity ability. Sensor. Actuator. B Chem..

[16] Kinnamon D., Ghanta R., Lin K.-C., Muthukumar S., Prasad S. (2017). Portable biosensor for monitoring cortisol in low-volume perspired human sweat. Sci. Rep..

[17] Tsoncheva T., Rosmini C., Dimitrov M., Issa G., Henych J., Nĕmečková Z., Kovacheva D., Velinov N., Atanasova G., Spassova I. (2020). Formation of catalytic active sites in hydrothermally obtained binary ceria–iron oxides: composition and preparation effects. ACS Appl. Mater. Interfaces.

[18] Manikandan V., Petrila I., Vigneselvan S., Mirzaei A., Mane R., Kim S.S., Chandrasekaran J. (2020). Enhanced humidity sensing properties of Fe-doped CeO 2 nanoparticles. J. Mater. Sci. Mater. Electron..

[19] Zhang L., Fang Q., Huang Y., Xu K., Ma F., Chu P.K. (2017). Facet-engineered CeO 2/graphene composites for enhanced NO 2 gas-sensing. J. Mater. Chem. C.

[20] Yuan K., Wang C.-Y., Zhu L.-Y., Cao Q., Yang J.-H., Li X.-X., Huang W., Wang Y.-Y., Lu H.-L., Zhang D.W. (2020). Fabrication of a micro-electromechanical system-based acetone gas sensor using CeO2 nanodot-decorated WO3 nanowires. ACS Appl. Mater. Interfaces.

[21] Jiang T., Wan P., Ren Z., Yan S. (2019). Anisotropic polyaniline/SWCNT composite films prepared by in situ electropolymerisation on highly oriented polyethylene for high-efficiency ammonia sensor. ACS Appl. Mater. Interfaces.

[22] Guo Z., Liao N., Zhang M., Xue W. (2018). Theoretical approach to evaluate graphene/PANI composite as highly selective ammonia sensor. Appl. Surf. Sci..

[23] Wu C., Han L., Zhang J., Wang Y., Wang R., Chen L. (2022). Capacitive ammonia sensor based on graphene oxide/polyaniline nanocomposites. Adv. Mat. Technol..

[24] Kroutil J., Laposa A., Voves J., Davydova M., Nahlik J., Kulha P., Husak M. (2018). Performance evaluation of low-cost flexible gas sensor array with nanocomposite polyaniline films. IEEE Sensor. J..

[25] Murugadoss G., Ma J., Ning X., Kumar M.R. (2019). Selective metal ions doped CeO2 nanoparticles for excellent photocatalytic activity under sun light and supercapacitor application. Inorg. Chem. Commun..

[26] Shah P.M., Burnett J.W., Morgan D.J., Davies T.E., Taylor S.H. (2019). Ceria–zirconia mixed metal oxides prepared via mechanochemical grinding of carbonates for the total oxidation of propane and naphthalene. Catalysts.

[27] Wang H., Tsilomelekis G. (2020). Catalytic performance and stability of Fe-doped CeO 2 in propane oxidative dehydrogenation using carbon dioxide as an oxidant. Catal. Sci. Technol..

[28] Kuzmanović B., Vujković M.J., Tomić N., Bajuk-Bogdanović D., Lazović V., Šljukić B., Ivanović N., Mentus S. (2019). The influence of oxygen vacancy concentration in nanodispersed non-stoichiometric CeO2-δ oxides on the physico-chemical properties of conducting polyaniline/CeO2 composites. Electrochim. Acta.

[29] Nakagawa K., Murata Y., Kishida M., Adachi M., Hiro M., Susa K. (2007). Formation and reaction activity of CeO2 nanoparticles of cubic structure and various shaped CeO2–TiO2 composite nanostructures. Mater. Chem. Phys..

[30] R. Anitha, E. Kumar, S.V. Durai, Structural, Optical, and Frequency Dependent Conductivity Properties of PANI/Ceo2 Nanocomposites by in Situ Polymerization Method.

[31] Hosseini M.G., Aboutalebi K. (2019). Enhancement the anticorrosive resistance of epoxy coatings by incorporation of CeO2@ polyaniline@ 2-mercaptobenzotiazole nanocomposite. Synth. Met..

[32] Nath B., Chaliha C., Kalita E., Kalita M. (2016). Synthesis and characterisation of ZnO: CeO2: nanocellulose: PANI bionanocomposite. A bimodal agent for arsenic adsorption and antibacterial action. Carbohydr. Polym..

[33] He Y. (2005). Synthesis of polyaniline/nano-CeO2 composite microspheres via a solid-stabilised emulsion route. Mater. Chem. Phys..

[34] Khan M.M., Khan W., Ahamed M., Alhazaa A.N. (2017). Microstructural properties and enhanced photocatalytic performance of Zn doped CeO2 nanocrystals. Sci. Rep..

[35] Li G., Smith R.L., Inomata H. (2001). Synthesis of nanoscale Ce1-x Fe x O2 solid solutions via a low-temperature approach. J. Am. Chem. Soc..

[36] Shen M., Wang J., Shang J., An Y., Wang J., Wang W. (2009). Modification ceria-zirconia mixed oxides by doping Sr using the reversed microemulsion for improved Pd-only three-way catalytic performance. J. Phys. Chem. C.

[37] Zhang Y.-W., Si R., Liao C.-S., Yan C.-H., Xiao C.-X., Kou Y. (2003). Facile alcohothermal synthesis, size-dependent ultraviolet absorption, and enhanced CO conversion activity of ceria nanocrystals. J. Phys. Chem. B.

[38] Wang W., Zhu Q., Qin F., Dai Q., Wang X. (2018). Fe doped CeO2 nanosheets as Fenton-like heterogeneous catalysts for degradation of salicylic acid. Chem. Eng. J..

[39] Wang Z.L., Feng X. (2003). Polyhedral shapes of CeO2 nanoparticles. J. Phys. Chem. B.

[40] Nallappan M., Gopalan M. (2018). Fabrication of CeO2/PANI composites for high energy density supercapacitors. Mater. Res. Bull..

[41] Liu C., Tai H., Zhang P., Yuan Z., Du X., Xie G., Jiang Y. (2018). A high-performance flexible gas sensor based on self-assembled PANI-CeO2 nanocomposite thin film for trace-level NH3 detection at room temperature. Sensor. Actuator. B Chem..

[42] Comini E., Faglia G., Sberveglieri G. (2001). UV light activation of tin oxide thin films for NO2 sensing at low temperatures. Sensor. Actuator. B Chem..

[43] Dhanawade R.N., Pawar N.S., Chougule M.A., Hingangavkar G.M., Jadhav Y.M., Nimbalkar T.M., Navale Y.H., Chavan G.T., Jeon C.-W., Patil V.B. (2023). Highly sensitive and selective PAni-CeO2 nanohybrid for detection of NH3 biomarker at room temperature. J. Mater. Sci. Mater. Electron..

[44] Berutti F., Alves A., Bergmann C., Clemens F., Graule T. (2009). Synthesis of CeO2 and Y2O3-doped CeO2 composite fibers by electrospinning. Part. Sci. Technol..

[45] Zhou J.Y., Bai J.L., Zhao H., Yang Z.Y., Gu X.Y., Huang B.Y., Zhao C.H., Cairang L., Sun G.Z., Zhang Z.X. (2018). Gas sensing enhancing mechanism via doping-induced oxygen vacancies for gas sensors based on indium tin oxide nanotubes. Sensor. Actuator. B Chem..

[46] Liu J., Dai M., Wang T., Sun P., Liang X., Lu G., Shimanoe K., Yamazoe N. (2016). Enhanced gas sensing properties of SnO2 hollow spheres decorated with CeO2 nanoparticles heterostructure composite materials. ACS Appl. Mater. Interfaces.

[47] Laguna O., Centeno M., Boutonnet M., Odriozola J.A. (2011). Fe-doped ceria solids synthesised by the microemulsion method for CO oxidation reactions. Appl. Catal. B Environ..

[48] Wang L., Huang H., Xiao S., Cai D., Liu Y., Liu B., Wang D., Wang C., Li H., Wang Y. (2014). Enhanced sensitivity and stability of room-temperature NH3 sensors using core–shell CeO2 nanoparticles@ cross-linked PANI with p–n heterojunctions. ACS Appl. Mater. Interfaces.

[49] Shankar P., Rayappan J.B.B. (2015). Gas sensing mechanism of metal oxides: the role of ambient atmosphere, type of semiconductor and gases-A review. Sci. Lett. J.

[50] Zhang D., Jiang C. (2017). Room-temperature high-performance ammonia gas sensor based on layer-by-layer self-assembled molybdenum disulfide/zinc oxide nanocomposite film. J. Alloys Compd..

[51] Mani G.K., Rayappan J.B.B. (2014). Selective detection of ammonia using spray pyrolysis deposited pure and nickel doped ZnO thin films. Appl. Surf. Sci..

[52] Espid E., Adeli B., Taghipour F. (2019). Enhanced gas sensing performance of photo-activated, Pt-decorated, single-crystal ZnO nanowires. J. Electrochem. Soc..

[53] Saboor F.H., Ueda T., Kamada K., Hyodo T., Mortazavi Y., Khodadadi A.A., Shimizu Y. (2016). Enhanced NO2 gas sensing performance of bare and Pd-loaded SnO2 thick film sensors under UV-light irradiation at room temperature. Sensor. Actuator. B Chem..

[54] Hu Q., Wang Z., Chang J., Wan P., Huang J., Feng L. (2021). Design and preparation of hollow NiO sphere-polyaniline composite for NH3 gas sensing at room temperature. Sensor. Actuator. B Chem..

[55] Dhanawade R.N., Pawar N.S., Chougule M.A., Hingangavkar G.M., Nimbalkar T.M., Chavan G.T., Jeon C.W., Patil V.B. (2023). Influence of the camphor sulphonic acid (CSA) intercalation on the micro‐structural and gas sensing properties of polyaniline‐CeO2 nanohybrid for NH3 gas detection. Chem. Select.

[56] Li P., Wang B., Qin C., Han C., Sun L., Wang Y. (2020). Band-gap-tunable CeO2 nanoparticles for room-temperature NH3 gas sensors. Ceram. Int..

[57] Bandgar D., Navale S., Naushad M., Mane R., Stadler F., Patil V. (2015). Ultra-sensitive polyaniline–iron oxide nanocomposite room temperature flexible ammonia sensor. RSC Adv..

[58] Khuspe G., Bandgar D., Sen S., Patil V. (2012). Fussy nanofibrous network of polyaniline (PANi) for NH3 detection. Synth. Met..

