# Supplementary material for: Preparation and characterisation of NH3 gas sensor based on PANI/Fe-doped CeO2 nanocomposite
Source: Heliyon. 2024 Jul 20;10(15):e34801. doi: 10.1016/j.heliyon.2024.e34801 (PMC11336279; doi:10.1016/j.heliyon.2024.e34801)
Supplement: Multimedia component 1 [file mmc1.docx]

| 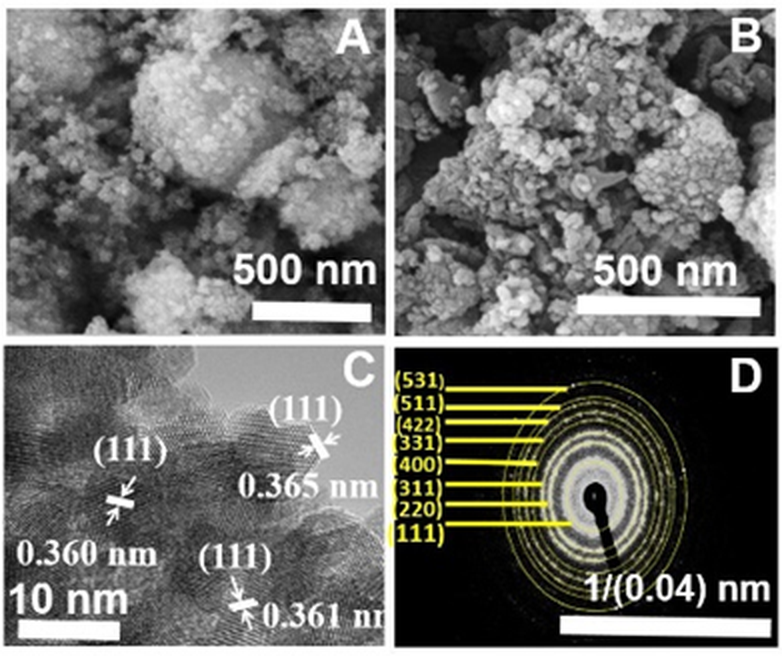 |
| --- |
|  |
| Fig. S1. A) and B) are the SEM images of CeO_2_ and CeO @PANI samples, C) and D) are the corresponding HRTEM and SAED patterns of sample A. |
